# HIV Prevention and Treatment Information from Four Artificial Intelligence Platforms: A Thematic Analysis

**DOI:** 10.1007/s10461-025-04786-9

**Published:** 2025-06-07

**Authors:** Stephen Beegle, Luis A. Gomez, Jason T. Blackard, Bingfang Yan, Jaime Robertson, Kevin T. Fedders, Shaina Horner, Ramsey Miller, Chavez R. Rodriguez, Abby Atreya, Jennifer L. Brown

**Affiliations:** 1 Department of Psychological Sciences, Purdue University, 703 3rd Street Room 1242, West Lafayette, IN 47907 USA; 2 Department of Internal Medicine, University of Cincinnati College of Medicine, Cincinnati, OH USA; 3 Center for Addiction Research, University of Cincinnati College of Medicine, Cincinnati, OH USA; 4 James L. Winkle College of Pharmacy, University of Cincinnati, Cincinnati, OH USA

**Keywords:** Artificial Intelligence (AI), Large Language Models, HIV Prevention and Treatment Health Information, Conventional content analysis, Thematic Analysis

## Abstract

Health information is highly accessible with the prominence of artificial intelligence (AI) platforms, such as Chat Generative Pre-Trained Transformer (ChatGPT). Within the context of human immunodeficiency virus (HIV), it is paramount to understand and evaluate the information being provided by AI platforms concerning the safety, side effects, and efficacy of medications to prevent and treat HIV. Prompts (*n* = 38) requesting information regarding HIV medication use for prevention and treatment were inputted into three AI-based Large Language Models (LLMs; ChatGPT 3.5, ChatGPT 4.0, Google Bard [now Gemini]) and one chatbot (HIV.gov Chatbot) on four consecutive weeks. Outputs (*n* = 608) were recorded verbatim, weekly by platform. Qualitative analyses using a conventional content analysis coding approach examined key themes in responses; response comprehensiveness was rated via the number of themes represented in a response. Core themes emerged across prompts. A recommendation to speak with a medical professional for further information was the most common theme across platforms. Organ/bone side effects were the most prevalent side effect. Responses pointed to medication efficacy to prevent and treat HIV. ChatGPT 4.0 provided the most comprehensive responses across platforms, while the HIV.gov Chatbot gave the least comprehensive information. Health information on HIV medication safety, side effects, and efficacy is widely available using AI platforms. Results indicate that AI responses typically included recommendations to consult a medical professional to personalize care. The efficacy of medications was never questioned across AI platforms. Future research directions for AI use within the context of HIV prevention and care are provided.

## Introduction

Artificial Intelligence (AI)-based Large Language Models (LLMs), such as Chat Generative Pre-Trained Transformer (ChatGPT) and Google Bard (now “Gemini”) provide fast and convenient access to personally tailored health information compared to other online health resources [1]. Unlike keyword-driven search engines that produce general results, LLMs provide contextually aware and personally curated information in response to search prompts. Although LLMs may offer great benefits to consumers, questions remain about the accuracy [2], sourcing [3], utility [4], and ethical application of LLMs for health-related purposes, especially for vulnerable populations [5]. One such patient population is individuals living with human immunodeficiency virus (HIV) or at risk for HIV, who require access to accurate and specialized care information for their health-related concerns. Sensitive health-related information directly delivered to people living with or at risk for HIV may affect individuals’ treatment decision-making. Further, it is important to assess the information provided by LLMs regarding the safety, side effects, and efficacy of medications to prevent and treat HIV. Thus, this study examined the content and comprehensiveness of HIV medication health information provided by four AI platforms.

Recent literature has evaluated the use of AI for health information distribution. Broadly, researchers have found that the quality of AI-delivered health information is of a similar caliber to information found on other medical websites [6] and to information provided by healthcare professionals [7]. Although AI-delivered health information may be of benefit to consumers, Liu et al. [4] warned that the lack of medical expertise and background may limit consumers’ ability to navigate complex treatment decisions and may reduce the reliability of the medical recommendations. Further, Al-Ashwal et al. [8] found that LLMs deliver variable levels of sensitivity, specificity, and overall accuracy in their ability to identify drug-drug interactions (DDI). However, a recent preprint by Most et al. [9] suggested that ChatGPT’s latest update (ChatGPT 4.0) demonstrated improvements in its ability to accurately identify DDI, but failed to reliably provide clinical recommendations that balance therapeutic benefit with risk of harm to the patient. Actionability of health information that can be used to accurately initiate treatment is lower for ChatGPT than both Google Search and treatment guidelines provided by health organizations (e.g., U.S. Food and Drug Administration [FDA]) [2]. However, there is limited literature differentiating between the actionability of different versions of ChatGPT (3.5 vs. 4.0) and other LLMs and chatbots, particularly for HIV-related content.

In the context of HIV prevention and treatment medication information, little research has evaluated the content and comprehensiveness of AI-delivered information. In one study that examined AI-delivered information regarding sexually transmitted infections (STI), ChatGPT failed to share relevant information about HIV pre-exposure prophylaxis (PrEP)– medications used to prevent the transmission of HIV [10]. Additionally, studies have reported evidence of bias when describing HIV-related information. For example, ChatGPT 4.0 presents information that does not adequately address racial discrimination, stigma, and healthcare disparities related to HIV [11] and may perpetuate myths that contribute to HIV stigma (e.g., suggestions that only gay men have anal sex; [12]). Finally, there have been few studies that have evaluated health information related to the efficacy, safety, and side effect profiles of medications to prevent and treat HIV. We identified one study that assessed ChatGPT’s ability to provide medication counseling for individuals initiating medications to treat HIV [13]. In this study, ChatGPT identified concerns regarding DDI and drug sensitivities related to the initiation of Triumeq^®^, a combination antiretroviral medication used to treat HIV [13]. The limited empirical evidence evaluating HIV medication information provided by AI platforms remains a critical gap in the literature.

The objective of this study was to examine the content provided by AI platforms regarding the availability of accurate, actionable information and the complexity of responses regarding HIV-related health information. Specifically, this study examined AI-delivered health information regarding the safety, efficacy, and side effects of antiretroviral medications used to prevent or treat HIV across four AI platforms. We examined: (a) core themes in responses, (b) the comprehensiveness of responses, (c) differences in the content provided between AI platforms, (d) within-platform longitudinal changes in content, and (e) the extent to which platforms provided references for the information they provided.

## Methods

### Procedures

The study employed a methodology adapted from Morath et al. [14] which examined drug-related information provided by ChatGPT via three independent raters. Across June and July 2023, 38 question prompts were input into four AI-based platforms (ChatGPT 3.5, ChatGPT 4.0, Google Bard, and HIV.gov Chatbot) on the same day of the week, once a week, for four weeks. Each question prompt was individually input in a new chat, and all questions and responses were collected and recorded verbatim by platform and by week. This process culminated in 608 responses (38 prompts × 4 platforms × 4 weeks). For this study, questions were designed to examine the safety, efficacy, and side effects of antiretroviral medications to prevent and treat HIV. Questions focused on: (i) general information on antiretroviral medication safety (e.g., “*Is Biktarvy® safe?*”), (ii) the efficacy of antiretroviral medications for the treatment of HIV (e.g., “*How well does Biktarvy*^*®*^*work?*”), (iii) the efficacy of antiretroviral medications for the prevention of HIV (“*How well does Descovy*^*®*^*work for PrEP?*”), and (iv) general information on antiretroviral medication side effects (e.g., “*What are the side effects of Descovy*^*®*^*for PrEP?*”). This study focused on tenofovir-based drugs, and prompts included both generic and brand names for the five most commonly used drugs (Biktarvy^®^, Descovy^®^, Genvoya^®^, Odefsey^®^, Truvada^®^), based on global pharmaceutical sales data [15].

### Data Analysis

To identify core themes in responses, qualitative analyses employed a conventional content analysis approach using NVivo software [16]. The analytic approach sought to identify core themes related to: (a) efficacy, (b) safety, and (c) side effects of medications used to prevent and treat HIV. Transcripts were annotated with notes and labels for potential coding themes and sub-themes related to HIV medication safety, efficacy, and side effects to develop the initial codebook. The initial codebook was used to code a randomly selected transcript with refinement of the initial coding classification scheme based on coding discrepancies and discussion of potential coding structure revisions using a standard iterative process [17, 18]. Two independent raters then coded all transcripts using the finalized codebook to apply codes representing each theme or sub-theme to text segments, with representative quotations identified. Coding discrepancies (i.e., differences in coded theme or sub-theme for segmented text) were resolved by a third coder. To determine response comprehensiveness, the total number of coded themes per platform was recorded, and higher values represented more comprehensive responses provided by the platform. Additionally, the consistency of response comprehensiveness was assessed across the four weeks of data collection.

## Results

### Core Identified Themes

Core themes were identified using a conventional content analysis approach. Themes highlighted: (a) the importance of consulting with a credentialed medical professional for personalized care advice; (b) provision of information from reputable domestic and global health organizations; (c) side effect information; (d) descriptive information about HIV medications; (e) information about medication usage; and (f) general medication safety and efficacy information. Each core theme is described below, and Table 1 provides representative responses for each AI platform for these core themes.

**Table 1:** Representative responses by theme across platforms

ChatGPT 3.5	ChatGPT 4.0	Google Bard	HIV.gov Chatbot
*Theme: Seeking advice from a medical professional*			
“It’s essential to consult with a qualified healthcare professional who can evaluate your specific medical condition and provide personalized advice on the safety and suitability of tenofovir for you. If you have concerns or experience any side effects while taking tenofovir, be sure to contact your healthcare provider promptly. Keep in mind that medical information may have evolved since my last update, so it’s crucial to refer to more recent sources and consult with a healthcare professional for the latest and most accurate information.” (Week 1)	“Given the complexity and the potential risks, it’s vital to consult with a healthcare provider who specializes in HIV prevention. The can assess your particular situation and determine if PrEP with tenofovir is the right strategy for you, considering your medical history, risk factors, and other individual circumstances.” (Week 3)	“If you are taking tenofovir, it is important to talk to your doctor about the risks and benefits of the medication. You should also be sure to get regular blood tests to monitor your kidney and liver function.” (Week 1)	“Talk to your health care provider about your side effects, and do not stop taking your HIV medicine or skip doses without first talking to your provider” (Week 1)
*Theme: Recommended information from a health organization*			
“For the most-up to date and accurate information regarding HIV medications and their safety, it is recommended to consult a healthcare professional or refer to reputable sources like the Centers for Disease Control and Prevention (CDC) or the World Health Organization (WHO).” (Week 3)	“Biktarvy has been approved by regulatory agencies like the U.S. Food and Drug Administration (FDA) and the European Medicines Agency (EMA) for specific uses in treating HIV” (Week 3)	“According to the U.S. Food and Drug Administration (FDA), Truvada is safe for most people when taken as directed.” (Week 4)	“A long-acting injectable form of PrEP, Apretude^®^, has also been approved by the FDA” (Week 2)
*Theme: Medication side effects*			
“**Side Effects**: Like most medications, HIV drugs may cause side effects. The type and severity of side effects can vary from person to person. Common side effects include nausea, diarrhea, fatigue, and headaches.” (Week 2)	“As of my knowledge cutoff in 2021, the most common side effects include diarrhea, nausea, fatigue, headache, dizziness, depression, insomnia, abnormal dreams, and rash.” (Week 1)	“The most common side effects of PrEP are mild and go away over time. These include nausea, diarrhea, headache, fatigue, and stomach pain. In some people, PrEP can also cause kidney or liver problems. However, these problems are rare, and they usually only occur in people who already have underlying health conditions.” (Week 2)	No example provided by HIV.gov chatbot
-“**Headache** Headaches are a possible side effect, although they are usually mild and temporary.” (Week 3)	-“**Headache.** This can be a common side effect but it usually mild.” (Week 3)	-“*** Headache.** Headache is a less common side effect of Genvoya. It usually goes away on its own, but you can take medication to help relieve it.” (Week 1)	No example provided by HIV.gov chatbot
-“1. Nausea” (Week 2)	-“1. Nausea or vomiting” (Week 1)	-“*Nausea” (Week 1)	“In some people, PrEP can cause side effects, such as nausea” (Week 3)
*Theme: Information regarding usage of HIV medications*			
“Odefsey is a medication used for the treatment of HIV-1 infection in adults and adolescents weighing at least 35 kg. It is a combination of three drugs: emtricitabine, rilpivirine, and tenofovir alafenamide.” (Week 3)	“Odefsey is a medication used to treat human immunodeficiency virus-1 (HIV-1) infection. Odefsey is used to manage HIV in certain adults and pediatric patients weighing at least 35kg (77 pounds) who are either new to antiretroviral therapy or who are virologically suppressed (HIV-1 RNA less than 50 copies per mL) on a stable antiretroviral regimen for at least six months with no history of treatment failure.” (Week 1)	“Odefsey is a highly effective HIV treatment. In clinical trials, it was shown to be able to suppress HIV to undetectable levels in over 90% of people who took it. This means that the amount of HIV in their blood was so low that it could not be detected by laboratory tests. Odefsey is also a convenient treatment. It is a single-table regimen, which means that you only need to take one pill once a day. This can make it easier to remember to take your medication and to stay on treatment.” (Week 1)	No example provided by HIV.gov Chatbot.
-“Truvada for PrEP has been shown to be highly effective when taken consistently and correctly. Clinical trials and real-world studies have demonstrated its effectiveness in reducing the risk of HIV transmission. When taken daily as prescribed, Truvada for PrEP can reduce the risk of HIV infection by more than 90% in various populations, including men who have sex with men, transgender woman, and heterosexual couples where one partner is HIV-positive.” (Week 4)	-“Pre-exposure prophylaxis (PrEP) medications, which include Truvada and Descovy, were generally considered safe for the prevention of HIV infection in at-risk individuals. These medications work by blocking an enzyme that the HIV virus needs to replicate itself, which helps to prevent the virus from establishing an infection if an exposure occurs.” (Week 1)	-“Truvada is a pre-exposure prophylaxis (PrEP) medication that is highly effective in preventing HIV infection. When taken as prescribed, Truvada can reduce the risk of getting HIV from sex by about 99%. This means that for every 100 people who take Truvada as prescribed, only 1 person is likely to get HIV.” (Week 1)	-“There are two oral medications approved for daily use as PrEP. They are combinations of two-anti HIV drugs in a single pill. The first is Truvada^®^ which is for all people at risk for HIV through sex or injection drug use. The second is Descovy^®^ which is for sexually active men and transgender women at risk of getting HIV” (Week 2)

*Seeking advice from a medical professional.* The most prevalent theme identified was seeking medical advice from a healthcare professional. Responses often contained a direct recommendation to consult with a healthcare professional (e.g., doctor, nurse practitioner, primary care physician, etc.). When asked about the safety or efficacy of the different medications, almost all responses across platforms emphasized the importance of speaking with a medical professional to further advice for specific needs as a patient. The recommendation to seek medical advice would always appear at the beginning or the end of the response as a foreword or reminder accompanying the response.

*Recommended information from a health organization.* A second theme across responses referred the user to health organizations and/or governing bodies that promote health information regarding HIV medications. The health organizations were the Centers for Disease Control and Prevention (CDC), the European Medicines Agency (EMA), the FDA, the World Health Organization (WHO), and ‘Other’ which encompassed a singular mention for any other agency. Responses would often reference these organizations when citing the provided information regarding safety, efficacy or side effects or as additional resources to provide additional information beyond the response. The most commonly referenced organization for additional guidelines or information was the FDA followed by the CDC. The EMA was mentioned more frequently by Google Bard than the other platforms. The HIV.gov Chatbot was primarily focused on providing references to the FDA and CDC.

*Medication side effects.* Across queries there were many commonly referenced side effects. The side effects most referenced across each platform were those related to organ or bone conditions, such as kidney and liver side effects or bone loss. Although these side effects were frequently mentioned across platforms, they were noted as rare side effects that usually affected those with other underlying or co-morbid health conditions. Gastrointestinal issues were also highly referenced across platforms; common medication side effects cited were nausea, diarrhea, and vomiting. Other common side effects were related to central nervous system symptoms such as dizziness and headaches. Less commonly noted side effects included fatigue and related symptoms (e.g., insomnia, drowsiness). The least referenced side effect across platforms was lactic acidosis.

The presentation of responses for side effects occurred in a multitude of ways that differed by individual query and platform. Responses across platforms stated the side effects in a bulleted list format, a descriptive paragraph, or a single statement with no additional context, all within the same week. The responses also differed with regards to the presentation of severe side effects, especially those that could be potentially fatal. Google Bard and ChatGPT 3.5 were more likely to describe medication side effects that might cause organ damage, whereas ChatGPT 4.0 would describe the symptoms and presentation of the organ-related side effects. The HIV.gov Chatbot provided the least amount of content and briefest descriptions of medication side effects, with a particular focus on gastrointestinal symptoms such as nausea, diarrhea and vomiting.

*Descriptive information about HIV medications.* This theme centered around information directly related to specific aspects of the medications, such as the medication’s composition, class, or the pharmaceutical company that produces the medication. Descriptions regarding the individual compounds that comprise the referenced medications were most common. These responses ranged from providing a list of the internal chemical composition (e.g., bictegravir, emtricitabine, tenofovir) to full explanations of the purpose of *each* component in preventing or treating HIV. The least commonly referenced medication information was the drug manufacturer and additional information about the drug’s development and testing. Information regarding the medication’s dosing information was also more commonly presented by Google Bard.

*Information about medication usage.* Many responses contained in-depth information about the medication’s intended purpose to either prevent HIV for populations at-risk of contracting HIV or for treatment among those living with HIV. Additionally, for PrEP-focused responses there was often a reference to the medication’s target population (e.g., men who have sex with men) and transmission type (e.g., injection drug use or sexual contact). Less commonly referenced were any additional factors that influenced the medication’s intended dosing information (e.g., weight range).

*Safety and efficacy.* Across AI platforms there was consistent agreement that HIV medications are safe for the purposes of both treatment and prevention. Notably, each platform was also uniform in its recommendations to consult additional resources in the form of medical professionals or reputable health organizations. These recommendations were present in response to questions about HIV medication safety broadly as well as questions about the safety of specific medications.

All AI platforms agreed that medications to prevent and treat HIV are generally safe, effective, and are crucial for managing HIV. Additionally, AI platforms agreed that Truvada and Descovy were highly effective in preventing HIV transmission when used as prescribed. However, there were multiple instances in which Biktarvy^®^, Genvoya^®^, and Odefsey^®^ were mistakenly identified as medications that had been approved and could be prescribed for PrEP. Table 2 provides examples of the variable presentation of safety information across platforms.

**Table 2:** Inconsistencies from thematic analysis

ChatGPT 3.5	ChatGPT 4.0	Google Bard	HIV.gov Chatbot
*Theme: Information about medication usage*			
“Odefsey is considered safe and effective for pre-exposure prophylaxis (PrEP) in certain populations.” (Week 4)	No example provided by ChatGPT 4.0	“Odefsey is a single-tablet regimen (STR) that is FDA-approved for pre-exposure prophylaxis (PrEP) to prevent HIV infection in adults and adolescents who are at high risk of HIV infection. In clinical trials, Odefsey was shown to be highly effective at preventing HIV infection. In one study, Odefsey was 99% effective at preventing HIV infection in men who have sex with men (MSM). In another study, Odefsey was 96% effective at preventing HIV infection in heterosexual women.” (Week 4)	No example provided by HIV.gov Chatbot
*Theme: Reference inclusion*			
No example provided by ChatGPT 3.5	No example provided by ChatGPT 4.0	“PrEP: Everything you need to Know: https://www.cdc.gov/hiv/basics/prep/about-prep.html” (Week 2)	“Here’s some info about PrEP medicines: https://www.cdc.gov/hiv/basics/prep/about-prep.html” (Week 1)

For each theme, differences in presentation of response consistency over time are presented

### Comprehensiveness and Consistency of Content Over time

**Fig. 1:**
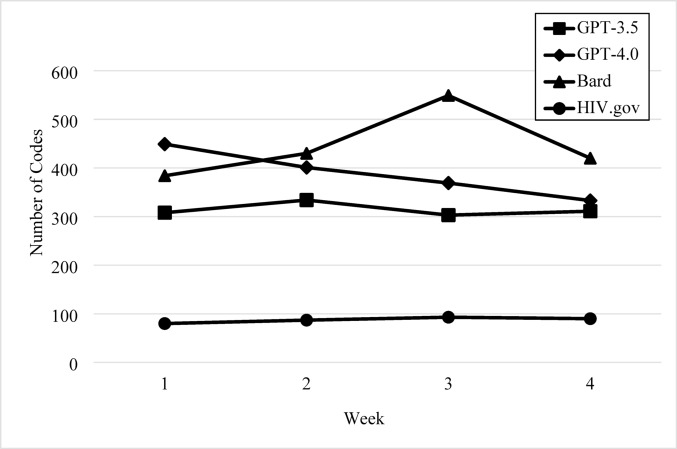
Comprehensiveness of Content over Time by AI Platform. There should be a separate note for the Figure that reads: Comprehensiveness of AI-generatedhealthcare information is shown for ChatGPT 3.5, ChatGPT 4.0, Google Bard, and HIV.govChatbot, measured by total number of codes per week

Across the four weeks of data collection and across prompts, Google Bard (1783 total codes) provided the most in-depth and comprehensive healthcare information on topics related to medications used to prevent and treat HIV, followed by ChatGPT 4.0 (1552 total codes), ChatGPT 3.5 (1256 total codes), and HIV.gov Chatbot (350 total codes). The trends in comprehensiveness across platforms are presented in Fig. 1.

AI platforms’ ability to deliver comprehensive information with consistency was assessed across time. HIV.gov Chatbot delivered healthcare information with the most consistency in its level of comprehensiveness followed by ChatGPT 3.5, ChatGPT 4.0, and then Google Bard, which produced the highest variability in the delivery of comprehensive content.

### Reference Inclusion

Citations for the information contained in the responses were only present in two of the platforms; Google Bard and HIV.gov Chatbot.^1^ Google Bard included citations with 22% of responses and HIV.gov Chatbot included citations with 72% of responses. The responses with references from Google Bard contained a hyperlink at the end of the response without directly referencing the information or source of the link. In contrast, the HIV.gov Chatbot frequently shared a hyperlink to referenced content and also acknowledged the link as an additional source for further information. Table 2 provides representative examples of the references provided in responses.

## Discussion

Although the use of LLMs for personalized health-related information is gaining momentum, questions about the information related to HIV treatment and prevention remain paramount to understanding if LLMs are accurate and reliable sources of information for consumers. This exploratory study examined healthcare information provided by four AI platforms regarding the safety, efficacy, and side effects of medications to treat and prevent HIV. Results highlighted variability in the comprehensiveness of information provided by LLMs regarding HIV medications.

Our examination of AI and chatbot responses revealed core themes across responses. Although there have been concerns about the accuracy of health information provided by AI, these results suggest that AI responses typically provide accurate information regarding HIV medication safety and efficacy. Additionally, responses commonly encouraged individuals to seek out specialized guidance from a medical professional and highlighted key information from domestic and international health organizations. Additional responses provided information about potential side effects and general information about the medications.

When seeking information related to HIV medications, LLMs overwhelmingly recommended that individuals consult with a licensed healthcare professional for additional information and personalized health treatment recommendations. The common presence of these recommendations indicates that the LLM algorithms are trained to direct individuals to consult with healthcare professionals who are either certified in or specialize in the provision of services to treat and prevent HIV. Thus, the consistent delivery of these recommendations is encouraging, as it suggests that these AI platforms were designed to communicate to users the limitations of the healthcare information provided by AI platforms and instead direct consumers to licensed professionals to reduce the potential for harm to individuals seeking to treat or prevent HIV transmission.

Google Bard and both models of ChatGPT provided in-depth information on medication side effects; the HIV.gov Chatbot provided very brief references to potential gastrointestinal symptoms and did not provide information on the remaining symptom categories. These platforms reported commonly cited side effects, which spanned six categories: gastrointestinal, central nervous system, fatigue, lactic acidosis, organ and bone effects, and other side effects. Google Bard reported medication side effects at a higher frequency than both ChatGPT models and the HIV.gov Chatbot. Organ/bone and gastrointestinal side effects were the most frequently cited side effects for the medications asked about in this study. Both ChatGPT models and Google Bard consistently provided warnings of the potential side effects associated with each medication, which also included allergy information and warnings about potentially serious or fatal side effects. Side effect warnings were frequently accompanied by recommendations to consult with a licensed healthcare professional and to visit the nearest emergency room in the event of serious adverse medication effects [19].

In large part, AI platforms accurately described the purpose of HIV medications. However, a few instances were observed in which platforms misrepresented and misclassified the purpose of a medication as HIV prevention when the drug was, in fact, solely designed to treat HIV in individuals already living with HIV. These inaccurate generated statements have been described as “AI hallucinations,” referring to instances when LLMs present statements that are factually incorrect, nonsensical, or inconsistent with established knowledge. This phenomenon has led researchers to publish warnings about the use of AI in healthcare [20, 21] and for which interventions are being researched [22, 23].

The findings of this study revealed consistency across AI platforms regarding the safety and efficacy of medications to treat and prevent HIV. ChatGPT 3.5, ChatGPT 4.0, Google Bard, and the HIV.gov Chatbot all consistently stated that medications to treat and prevent HIV were considered safe and efficacious. These findings are encouraging, as they indicate an alignment between AI-generated health information and previously established research on the safety and efficacy of Descovy^®^ [24, 25], Truvada^®^ [26], Biktarvy^®^ [27, 28], Genvoya^®^ [29, 30], and Odefsey^®^ [31, 32].

Although both ChatGPT models and Google Bard generated in-depth, comprehensive information regarding HIV medications for prevention and treatment, Google Bard was the least consistent across time regarding the information it generated. In other words, Google Bard demonstrated the largest variability in its coverage of various healthcare topics. These AI platforms provided the most comprehensive information about the purpose and side effects of each HIV medication and consistently provided recommendations to consult with healthcare professionals. In contrast, the HIV.gov Chatbot provided the shortest responses, covered far fewer topics, and provided the most limited and narrow scope of information across healthcare topics compared to ChatGPT 3.5, ChatGPT 4.0, and Google Bard, which could be attributed to the robust LLMs that make both ChatGPT models and Google Bard advanced platforms for large language processing. This finding is consistent with research by Al-Ashwal et al. [8] in which multiple AI platforms demonstrated variable ability to identify and discuss key information when asked about medication interactions.

The HIV.gov Chatbot frequently provided citations and hyperlinks to additional information, while Google Bard provided citations for a small proportion of its responses. Neither ChatGPT 3.5 nor ChatGPT 4.0 provided citations. This finding is concerning, as consumers would have no way of verifying the validity of the information generated by these AI platforms. A recent report published by Columbia Journalism Review found that AI chatbots often confidently provided inaccurate or speculative answers and often failed to properly link to the original source, in some instances citing fabricated or broken URLs [33]. Future research should further examine the nature of the referenced content and the credibility of referenced sources. Additionally, as LLMs evolve and users can request responses providing references, this content should be scrutinized.

This study has notable strengths that increase its applicability to future research. First, the study employed a well-established content analysis qualitative design in which the codes applied by two independent raters were resolved by a third independent coder. Second, the study’s longitudinal design allowed us to assess the consistency of each AI platform, wherein we examined the degree to which AI platforms delivered the same breadth of information in response to the same prompt input across multiple trials (once a week over a four-week period).

There were several limitations to our study that future research can address. First, rapid advancements and updates to LLM-based AI platforms may have rendered some of our findings outdated. For example, at the time of this manuscript submission, ChatGPT 3.5 was no longer in use, ChatGPT 4.0 became the default free version (featuring online search capabilities that include referenced sources), and Google Bard was replaced by Google Gemini. Similarly, there have been platform changes regarding the provision of cited references. For example, recent studies have found that ChatGPT 4.0 frequently provided inaccurate or nonexistent references [34–36]. In contrast, one study found that all citations provided by ChatGPT 4.0 were real and accurate [37]. Notably, Google Bard’s successor, Gemini, has been found to consistently provide accurate references [36, 38]. Future research in this area will necessitate accelerated research methodologies to evaluate content provided by LLM-based AI platforms. Second, only four LLM platforms were used despite many different possible options. ChatGPT paid version (GPT 4.0), although a different LLM than ChatGPT free (GPT 3.5), is still part of the same company and therefore could share the same approach to knowledge acquisition and could further narrow the scope of the study. Third, how the questions were phrased may vary from the manner in which the average member of the public would ask, which could lead to less applicable results for generalization. Researchers could potentially “crowdsource” questions from individuals who work outside of the academic or medical domains and test those with LLMs. Fourth, specific keywords in the question inputs seemed to be valued by the LLMs as being more relevant to the question output and would sometimes lead to the LLM ignoring or misinterpreting the meaning of the question that was inputted. Fifth, content is updated over time at set scheduled update points rather than longitudinally which would have altered the initial data collection approach. This may serve as an avenue to conduct between-version research to assess changes to the information provided by LLMs. However, the nature of updates and associated implications (e.g., effect on the algorithm) may not be fully transparent to the end user. Sixth, despite referring to guidance from reputable health organizations (e.g., CDC, FDA, WHO), AI platforms demonstrated bias toward sources published in English from Western contexts, potentially limiting the generalizability of AI platforms’ responses to users who are not native English speakers or from a Western background. Future research should focus on determining the most consistent ways that LLMs can positively contribute as a tool in healthcare with their ever-increasing prevalence globally.

## Conclusions

This study explored the information that LLMs provided regarding the safety, efficacy, and side effects of medications used to treat and prevent HIV. The results of this study suggest that LLMs are reliable sources for comprehensive and in-depth information related to HIV medications. Google Bard provided the most comprehensive information on medication side effects. Further, LLMs approached HIV healthcare information with sensitivity. The HIV.gov Chatbot was the least informative platform for HIV medication information but frequently provided citations and links to additional information. LLMs need to improve the frequency with which they provide references to verifiable sources of information so that consumers may assess the validity of the information they receive from LLMs. LLMs provided accurate HIV medication information and also encouraged consulting with licensed healthcare professionals for the latest information that can be personally tailored to their HIV treatment or prevention needs. In the rapidly evolving landscape of AI for delivery of HIV-related health information, continued research is needed to examine the strengths and limitations of this technology.

## Footnotes

1 As of May 2025, ChatGPT offers a “Search” function that provides online references in its responses, which is off by default when a new thread is opened. The authors address the inclusion of references by AI platforms in more detail in the study limitations.
